# Unravelling the lung cancer diagnostic pathway: identifying gaps and opportunities for improvement

**DOI:** 10.2478/raon-2024-0025

**Published:** 2024-04-14

**Authors:** Mateja Marc Malovrh, Katja Adamic

**Affiliations:** University Clinic for Respiratory and Allergic Diseases Golnik, Golnik, Slovenia; Faculty of Medicine, University of Ljubljana, Ljubljana, Slovenia

**Keywords:** lung cancer, diagnostic pathway, improvement

## Abstract

**Background:**

A fast and well-organized complex diagnostic process is important for better success in the treatment of lung cancer patients. The aim of our study was to reveal the gaps and inefficiencies in the diagnostic process and to suggest improvement strategies in a single tertiary centre in Slovenia.

**Patients and methods:**

We employed a comprehensive approach to carefully dissect all the steps in the diagnostic journey for individuals suspected of having lung cancer. We gathered and analysed information from employees and patients involved in the process by dedicated questionnaires. Further, we analysed the patients’ data and calculated the diagnostic intervals for patients in two different periods.

**Results:**

The major concerns among employees were stress and excessive administrative work. The important result of the visual journey and staff reports was the design of electronic diagnostic clinical pathway (eDCP), which could substantially increase safety and efficacy by diminishing the administrative burden of the employees. The patients were generally highly satisfied with diagnostic journey, but reported too long waiting times. By analysing two time periods, we revealed that diagnostic intervals exceeded the recommended timelines and got importantly shorter after two interventions - strengthening the diagnostic team and specially by purchase of additional PET-CT machine (the average time from general practitioner (GP) referral to the multidisciplinary treatment board (MDTB) decision was 50.8 [± 3.0] prior and 37.1 [± 2.3] days after the interventions).

**Conclusions:**

The study illuminated opportunities for refining the diagnostic journey for lung cancer patients, underscoring the importance of both administrative and capacity-related enhancements.

## Introduction

Lung cancer remains a leading cause of cancer-related morbidity and mortality worldwide. Many patients are diagnosed at advanced stages of the disease.^1,2^ Despite advances in treatment modalities, early detection, and our understanding of the molecular aspects of oncology, we still face challenges in improving patient care and outcomes.^3,4^ Specifically, issues within the diagnostic process can lead to delayed diagnosis, treatment, and ultimately worse outcomes for lung cancer patients.^5,6^ Previous studies have shown that the complexities of healthcare systems and disjointed care impact lung cancer patients.^7^ Factors such as limited access to specialized services, especially for those in rural or remote areas, along with the need for coordinated care among various healthcare providers, result in delays and inefficiencies during diagnosis.^8^ Although the implementation of standardized care pathways has been proven to improve patient outcomes and satisfaction, their application varies across cancer types, settings, and populations.^9,10^ Our study aimed to address these issues and enhance lung cancer care. We planned to analyse gaps in the diagnostic process for individuals suspected of having lung cancer at University Clinic of Respiratory and Allergic Diseases Golnik. Research from different regions and healthcare settings gave us the starting idea for the research.^11,12^ Our work aims to comprehend the obstacles to timely and effective lung cancer diagnosis and suggest improvement strategies. By thoroughly assessing the diagnostic journey, our study delved into the vital aspects of lung cancer care paths. We identified factors that aid or hinder their implementation and assessed their impact on patient outcomes. Ultimately, our findings will be able to guide the creation of customized lung cancer care pathways that cater to our population’s and healthcare system’s specific needs. The objective was to elevate the quality and efficiency of care for individuals with suspected lung cancer.

## Patients and methods

To conduct this study, we employed a comprehensive approach to analyse the diagnostic journey for individuals suspected of having lung cancer. We undertook the following steps to ensure a thorough understanding of the process:

### Visual representation of the patient journey

We created a visual representation of the patient journey with input from an interdisciplinary team. The team included interventional pulmonology specialists (involved in triage, outpatient exams, (day)hospital work, and invasive procedures such as bronchoscopies and pleural punctures), radiologists for CT consultations and transthoracic biopsy guidance, pathologists, nurses in outpatient and inpatient settings, and an administrator and coordinator responsible for administrative tasks like issuing discharge letters and forwarding delayed reports from imaging or pathology investigations (especially PET CT or MRI performed in other institutions) to physicians. The patient journey illustration showed the stages and steps in the diagnostic process and interactions among healthcare providers. The key organizational characteristics of a clinic that diagnoses one-third of Slovenian patients with lung cancer are listed below:

- Patients with suspected lung cancer were managed in a specialized multidisciplinary tertiary center offering a range of necessary examinations, excluding PET-CT and MRI.
- Many examinations were conducted on an out-patient basis, making it ideal for identifying and preparing patients for invasive diagnostics, which were performed in hospitalized patients.
- A proficient triage system was established, involving a coordinator, interventional pulmonologist, and radiologist. The coordinator managed referrals, provided patient information, and organized outpatient exams or hospital admissions. The interventional pulmonologist and radiologist determined the need for further invasive diagnostics.
- Ideally, patients had outpatient exams before invasive diagnostics. Patients with prior out-patient exams were scheduled for invasive procedures (e.g., bronchoscopy, CT or US-guided transthoracic biopsy, US-guided lymph node aspiration, thoracentesis) on admission day, often as day-hospital cases.
- Directly admitted patients underwent invasive diagnostics the following day.
- The diagnostic process concluded by presenting patient data to the multidisciplinary treatment board (MDTB) for decision-making, communicating treatment choices to the patient, and scheduling lung cancer specialist follow-up.

### Patient data collection and analysis

After obtaining their written consent, we analysed data from patients referred to the clinic with suspected lung cancer from January 1, 2023, to March 31, 2023. Information was gathered from the hospital’s triage list, recording key events (date of referral, date of first / second appointment), triage decision, and methods of management (first visit at outpatient department, direct hospital admission, redirection to other facilities). Additional data on patients referred in January 2023 were extracted from the hospital information data system, including age, sex, final diagnosis, hospitalization duration, invasive procedures performed and MDTB treatment decision. Diagnostic intervals for hospitalized individuals with suspected lung cancer referred in January and June 2023 were computed from an excel spreadsheet and the Hospital Information system. All individual data were anonymized and in accordance with the General Data Protection Regulation. Ethical approvals were obtained from the Medical Ethics Committee of the Republic of Slovenia Nr. 0120-317/2016/2.

### Hospital staff survey

An online survey was administered to hospital staff to gather further insights and perspectives on the diagnostic patient journey and identify potential areas for improvement (Supplementary Table 1).

### Patient questionnaires

Four questionnaires (Q1−4) were designed especially for this survey to capture patient perspectives at different diagnostic journey stages:

g. Q1 - referral by the general practitioner (GP) to acceptance at the outpatient clinic (Supplementary Table 2).;
h. Q2 - after the outpatient clinic visit (Supplementary Table 3).;
i. Q3 - following hospitalization with an invasive diagnostic procedure (Supplementary Table 4).;
j. Q4 - after receiving a diagnosis (Supplementary Table 5).

After obtaining their written consent, the patients completed the questionnaires anonymously via the iPad they received at the hospital visits (Q1–Q3). The answers for Q4 questionnaire were obtained by phone call one week after the final diagnosis by medical students.

### Validation workshops

The accuracy and comprehensiveness of the patient journey map was validated through two workshops involving interdisciplinary team members who had previously contributed insights. These workshops facilitated discussions and feedback to refine the patient journey representation.

## Results

### Analysis of patients’ data from triage and hospitalized patients with lung cancer

**FIGURE 1.**
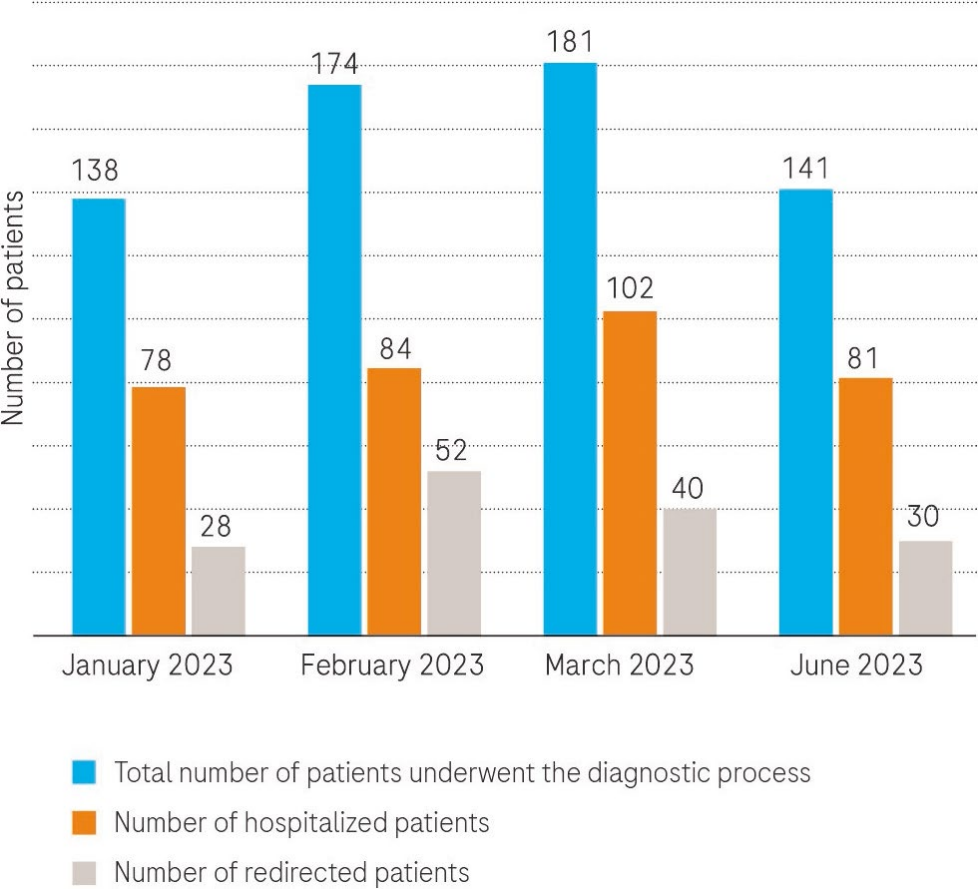
Triage for patients with lung infiltrates from January 1 to the end of March 2023 and in June 2023 (total number of referred patients, number of hospitalized patients, and number of redirected patients per month).

The analysis of the three-month period (from January 1, 2023, to March 31, 2023) revealed that a total of 493 patients underwent the diagnostic process for lung infiltrates. On average, this accounted for 164 patients per month (ranging between 138 and 181). Among the referred patients, 120 individuals (24.3%) were redirected to alternative facilities following the initial triage, which involved chest X-rays, CT scans and medical documentation assessment. This redirection occurred due to the absence of suspicion for malignant disease. Of all the patients referred, 264 (53.8%) required hospitalization for invasive diagnostics. In June 2023, out of the 141 referred patients, 30 individuals (27%) were redirected to other facilities after the initial triage. Furthermore, 82 patients (58.2%) needed hospitalization for further diagnostic procedures (Figure 1).

### Hospitalized patients from triage in January 2023

A detailed analysis was conducted on a subgroup of 75 patients who were referred for diagnostic evaluation in January 2023 and necessitated hospitalization. Within this subset, the final diagnoses encompassed various categories, with 39 patients (representing 52% of the hospitalized individuals and 28% of the referred patients) receiving a diagnosis of lung cancer, 9 cases involving lung metastases originating from other primary cancers, 16 patients with benign lesions, and 11 cases involving pleural diseases. Mean age of 39 patients with lung cancer was 70.0 years (± 1.3 Standard Error of the Mean [SEM]), 15.4% were older than 80 years, 7.7% younger than 60 years. Out of this group, 23 were male (59%). On average, their hospital stay spanned 2.6 (± 0.3) nights, with five individuals managed as day-hospital cases. Within this subgroup, 18 patients were categorized as stage I (46.2%), 5 as stage II (12.8%), 7 as stage III (17.9%), and 9 as stage IV (23.1%), based on disease TNM staging. The mean number of invasive procedures conducted per patient was 1.4 (± 0.09). Specifically, 19 patients underwent solely bronchoscopy, 12 received a combination of bronchoscopy and transthoracic needle aspiration (TTNA), two patients underwent only TTNA, three patients underwent ultrasound-guided peripheral lymph node puncture, and one patient underwent thoracentesis. Additionally, circulating DNA in plasma (ctDNA) analysis was performed in 2 patients with poor performance status. The MDTB recommendations varied among the patients, with 17 individuals advised to undergo surgery, 7 recommended for radical radiotherapy, 5 recommended for radical radiotherapy with concomitant systemic treatment, 5 prescribed systemic therapy, and five patients deemed suitable for best supportive care.

### Diagnostic intervals for patients with lung cancer

We calculated the time it took to diagnose lung cancer patients referred to our clinic in January 2023 and in June 2023. We selected these two time periods because they coincided with two significant changes that impacted the diagnostic process. First, a new PET CT machine was acquired (the fourth in Slovenia), and second, organizational changes were implemented within the clinic. These organizational changes included strengthening the day-hospital operations and involving other wards in the reception of lung cancer patients from the waiting list, resulting in a 50% increase in our hospital’s capacity to manage lung cancer patients.

Here are the key findings from our analysis:

For the 39 hospitalized lung cancer patients referred in January 2023:

- The average time from GP referral to the first examination in the clinic was 17.9 (± 0.9) days.
- The average time from GP referral to the final diagnosis (MDTB treatment decision) was 50.8 (± 3.0) days.
- 25% of patients received their first clinic appointment in under two weeks, and 20% received their final diagnosis within the recommended 31 days.
- The mean waiting time for a PET CT scan was 28.3 (± 3.4) days.

For the 38 hospitalized lung cancer patients referred in June 2023:

- The average time from GP referral to the first examination in the clinic was 13.6 (± 0.9) days.
- The average time from GP referral to the final diagnosis was 38.1 (± 2.3) days.
- 60% of patients received their first clinic appointment within two weeks, and 31.5% received their final diagnosis within the recommended 31 days.
- The mean waiting time for a PET CT scan was 19.5 (± 2.8) days.

### Hospital staff survey results

The results from a survey (Supplementary Table 1) conducted among 13 responsive team members, comprising four pulmonology specialists, one coordinator, one administrator, and seven nurses, have provided valuable insights into the work conditions within our hospital.

Here are the key findings:

- Work overload: A significant 90% of respondents expressed that they felt overloaded with their workload.
- Multiple workplace demands: Many staff members also faced challenges related to concurrent work across various hospital departments on the same day (ward, emergency department, bronchoscopy, outpatient pulmonology department, student tutoring). This multitasking affected their ability to dedicate sufficient time to patient discussions, explanations of the diagnostic procedures, final diagnoses, and treatment plans, particularly in outpatient settings and during brief hospital encounters.
- Job satisfaction: The majority of participants reported satisfaction with their workplace, appreciating the responsible, non-monotonous, and meaningful nature of their roles.
- Stress levels: All respondents reported experiencing at least moderate levels of stress, with 25% indicating severe stress at work.

Challenges identified:

- Patient care: The majority pulmonology specialists highlighted the challenges of managing numerous patients at various stages of the diagnostic process. They expressed dissatisfaction with the time-consuming system for tracking the newly arrived results of already discharged patients. The existing information system lacked alerts regarding patients not presented to the MDTB or the completion of their diagnostic path. To mitigate delays and potential loss of patient documentation, an Excel table was introduced in the past to track patients’ progress in the diagnostic pathway, including test results from pathology or radiology departments and presentation dates to the MDTB council (only for cases where such evaluation is needed). However, this process was found to be time-consuming and prone to inconsistencies and errors.
- Process duration: The extended duration of the entire diagnostic process was a concern.
- Administrative burden: Redundant administrative tasks were cited as a significant issue.
- Time pressure: The awareness that time constraints could impact patient outcomes added to their stress.
- These survey results shed light on the need for targeted interventions to alleviate workload pressures, streamline administrative tasks, and enhance the quality of patient care and communication within our hospital.

### Patient questionnaire results

We collected responses from patient questionnaires (Supplementary Tables 2−5) at various stages of their patient journey. Here is a breakdown of when and how many of these questionnaires were administered:

- Questionnaire 1 (Q1, Supplementary Table 2): Administered to 52 patients upon their arrival at the outpatient clinic in September 2022.
- Questionnaire 2 (Q2, Supplementary Table 3): Administered to the same patients after they completed their outpatient examination.
- Questionnaire 3 (Q3, Supplementary Table 4): Collected from 47 patients at the end of their hospitalization, spanning two time periods, in September–October 2022 and May–June 2023.
- Questionnaire 4 (Q4, Supplementary Table 5): Gathered from the same patients as Q3 approximately one week after their presentation to MDTB council, during the mentioned time periods.

Patient response rates vary across questions, and the number of respondents for each question is indicated next to the corresponding figure (see the N numbers listed with each Figure).

**FIGURE 2.**
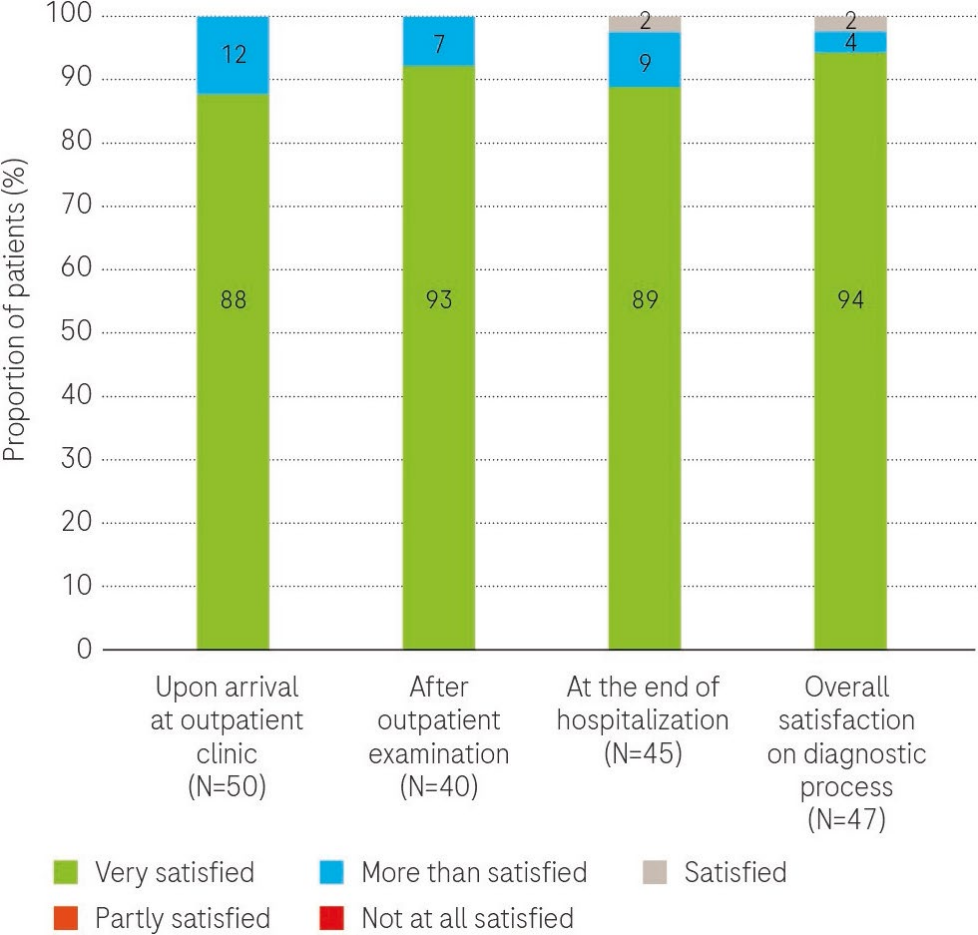
Patient satisfaction across diagnostic process stages. The proportion of patients who were asked: How satisfied were you during the diagnostic process? and answered with 1 (not satisfied) to 5 (very satisfied).

The majority of patients expressed high levels of satisfaction with all aspects of their experience throughout the diagnostic process for lung cancer, encompassing the period from their initial visit to the outpatient clinic to their discharge and the subsequent waiting period for the MDTB decision, as well as the receipt of the MDTB treatment decision information (as illustrated in Figure 2).

These results underscore the positive feedback received from patients regarding both the organizational and professional aspects of their diagnostic journey.

In our evaluation of the patient experience, we found the following key points:

- Outpatient medical check-ups: Medical checkups in the outpatient department were quick, with all patients completing them in under 30 minutes. A substantial 63% of patients finished their check-ups in under 15 minutes. All patients were satisfied with the information provided by the medical staff, with the exception of one patient who missed PET-CT information.
- Hospital admissions: Among hospitalized patients, the breakdown was as follows:
  - 40% were admitted after check-up in the out-patient department.
  - 42% were triaged for direct admission.
  - 18% with acute symptoms bypassed triage and were admitted through the emergency unit.

For 80% of hospitalized patients, a bed was available in less than 1 hour, while for the remaining 20%, it took up to 2 hours.

**FIGURE 3.**
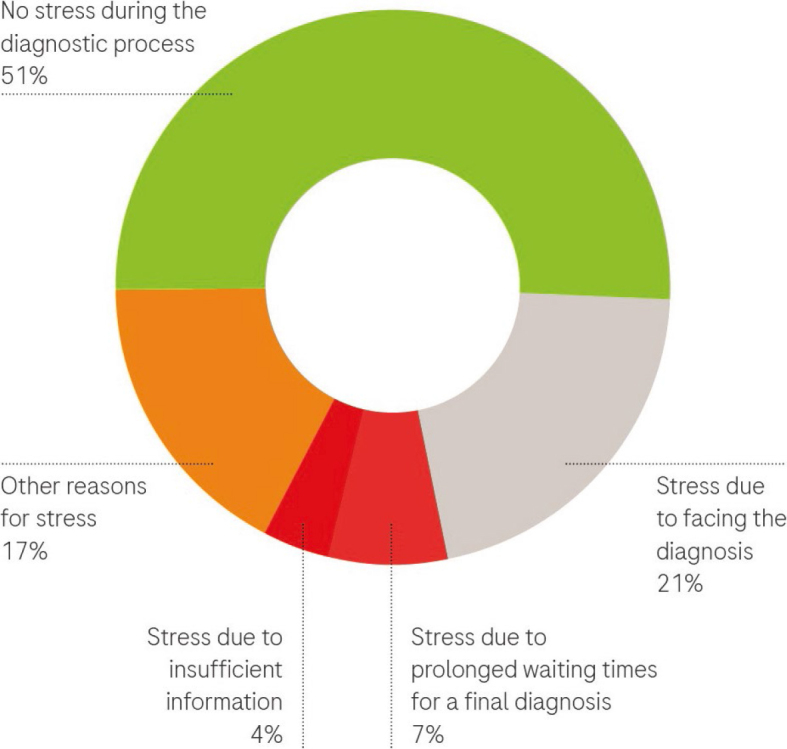
Sources of stress among patients during the diagnostic process (N = 47). Other reasons were sharing a room with three patients, Covid-19 infection, communication issues with staff, feelings of helplessness, social concerns, and fear.

**FIGURE 4.**
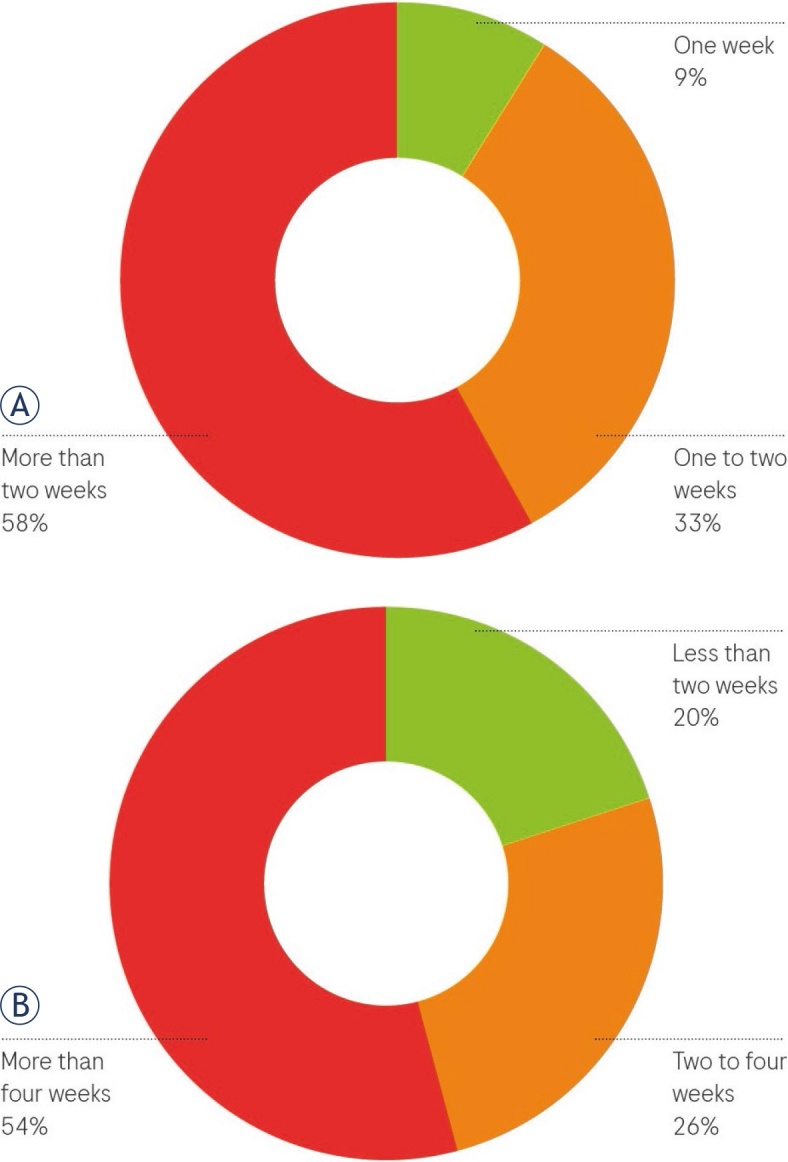
Patient reported waiting times from the GP referral to an initial appointment at the Golnik University Clinic (N = 52) **(A)**; and for receiving a final diagnosis **(B)**.

- Information and communication:
  - 89% of patients felt they received sufficient information from healthcare professionals
  - 20% expressed a desire for more time to converse with the doctor.
  - Over 90% believed they could understand information about their illness and knew who to contact for additional questions.
- MDTB decision communication: The information about the MDTB decision was effectively conveyed to the majority of patients, through either phone calls or in-person conversations. Patients comprehended the information and were aware of the subsequent steps. Relatives were also adequately informed.
- Stress levels: 51% of patients reported no stress during the diagnostic process. The remaining patients experienced stress due to various reasons, including: facing the diagnosis, prolonged waiting times for a final diagnosis, insufficient information, other factors such as sharing a room with three patients, Covid-19 infection, communication issues with staff, feelings of helplessness, social concerns, and fear. In Figure 3, we illustrate the proportions of patients reporting stress due to different reasons during their diagnostic journey.
- Patient-reported waiting times: we have provided detailed data on patient-reported waiting times for major events in their diagnostic journey in Figure 4A and 4B.
- Key findings from the data include:
  - Over 50% of patients reported waiting for more than 2 weeks for their initial appointment at the clinic, whether it was for an out-patient examination or hospital admission.
  - Similarly, for the period from GP referral to the receipt of a final diagnosis, more than 50% of patients reported waiting times that exceeded the recommended four-week period.

### Validation, data analysis and opportunities for improvement

Upon conducting a comprehensive assessment that included visual mapping of the patient journey and analysis of both hospital staff and patient surveys, several challenges and potential enhancements have become known. These improvements have the potential to elevate satisfaction levels and reduce stress for all involved parties.

#### Organizational challenges

- Patient tracking: Currently, patient tracking is managed simultaneously through Excel spreadsheets, which places an additional administrative burden on physicians and increases the likelihood of errors.
- Redundant administrative work: We have identified redundant administrative tasks, including the maintenance of patient records in both the Hospital Information System and separate files.
- Transcription of dictated notes: Administrative resources are dedicated to transcribing doctors’ dictated notes, resulting in inefficient use of work force.
- MDTB data organization: The organization of patient data required for the MDTB is sub-optimal, necessitating additional administrative work for documentation preparation, although most data are accessible in the information system.

In response to these challenges, we designed an electronic diagnostic clinical pathway (e-DCP), which could be incorporated in the existing clinical informational system. The e-DCP’s structure would encompass predefined options for various investigations, ensuring precise tracking of each patient’s progress within the diagnostic journey. Additional administrative efficiency gains could be realized by automating the generation of a concise summary of patient essential information extracted from the Electronic Health Record (EHR) for the MDTB proceedings. Notably, the system is designed to promptly flag any gaps or missing information and alert the physician when the patient is prepared for MDTB presentation.

#### Patient-centric challenges

- Lack of systematic patient feedback: Currently, patient feedback is not systematically collected, potentially overlooking valuable insights from the patient perspective.
- Communication channels: Patient communication with the hospital is limited to postal mail, telephone, or email. Exploring additional digital communication channels could enhance the patient experience.
- Patient awareness: Patients often lack explicit information about their current position within the patient journey and are unaware of the expected next steps.

Based on our analysis, data from two different periods, and patient reports, it is evident that in the majority of cases, the final diagnosis exceeds the recommended 31-day timeframe. This is primarily attributed to extended waiting times from referral to the first appointment and the challenge of limited PET-CT machines, resulting in lengthy waiting periods. Addressing these organizational and patient-centric challenges, as well as streamlining the diagnostic process, will be essential to improve the overall experience for patients and hospital staff alike.

## Discussion

This study takes a comprehensive approach to enhance our understanding of the diagnostic trajectory for patients with lung cancer, focusing on patient and medical staff experiences at a tertiary care center and pre-treatment temporal intervals. To our knowledge, this is the first in-depth survey of patient and medical staff experiences during the diagnostic path of lung cancer in tertiary centers.

Previous study by Rankin NM and colleagues provided insights into how patients with suspected lung cancer and their general practitioners (GPs) experience the diagnostic journey. Their findings highlighted that the lack of defined diagnostic pathways to respiratory specialist assessment and hospital clinics was a clear source of frustration for both patients and GPs. They recommended the implementation of national lung cancer pathways, which have shown to improve outcomes for lung cancer patients and may help address GP frustrations and health system barriers.^13^ However, our case differs from Rankin’s study, as our lung cancer diagnostic path is well defined, offering a simple and uniform process of referring patients with suspected lung cancer to a tertiary diagnostic center for all GPs and pulmonologists, regardless of the region. Additionally, our tertiary multidisciplinary diagnostic center operates with a well-coordinated and structured diagnostic pathway. These factors likely contribute significantly to the high rates of patient satisfaction and surprisingly low reported stress levels in our setting. Despite the well-structured and coordinated diagnostic pathway for lung cancer, our investigation revealed critical areas in need of improvement. These potential enhancements primarily pertain to either hospital staff or patients.

The findings from an online survey among hospital staff have highlighted two significant areas of concern: stress and excessive administrative workload. Stress primarily results from the limited time available to manage patient care. Additionally, extended timeframes until the final diagnosis and deficiencies in administrative patient tracking contribute to stress and frustration. To address these issues, the most frequently proposed improvements include: (1) Streamlining administrative tasks to reduce workload; (2) Implementing a system for regular and automated notification of test results after a patient’s discharge from the hospital; and (3) Enhancing access to specific medical services, such as hospital admissions for invasive diagnostics, PET-CT scans and MRIs. These measures have been suggested as means to alleviate stress and enhance the overall efficiency of patient care.

Significant strides in administrative patient management can be achieved by seamlessly integrating an electronic Diagnostic Clinical Pathway (e-DCP) into the existing Hospital Information System. The e-DCP concept envisions predefined options and a comprehensive patient tracking system that spans all diagnostic stages, from initial triage to the final diagnosis and presentation to MDTB for treatment decisions. We have carefully designed this e-DCP, and its implementation is currently pending. This automation would significantly reduce the present workload associated with documentation preparation. The ultimate goal of e-DCP implementation is to entirely eliminate the need for paper records and the current Excel spreadsheet-based patient tracking system.

Furthermore, once the patient data summary is systematically organized, it should be automatically integrated into both the Clinical and National Lung Cancer Registry, further streamlining the process and enhancing data accuracy.

Further, in the current scenario, considerable administrative resources are dedicated to transcribing physicians’ dictated notes. This inefficiency could be significantly streamlined by embracing appropriate Speech Recognition technology, designed to directly transcribe doctors’ notes into the Patient’s Electronic Health Record (EHR). Ideally, this application would convert unstructured text into a standardized format, enhancing overall efficiency and accuracy.

A significant drawback in the process was the extended timeframe from the initial referral to the final diagnosis, a factor that also contributed significantly to stress among both patients and medical staff. The influence of diagnostic process speed on patient outcomes and survival has been extensively explored in the literature.^12,14,15,16,17,18^ These studies have yielded mixed results due to the high heterogeneity of patients and variations in diagnostic pathways, which often prioritize faster evaluation for more severe cases. Nonetheless, it is widely acknowledged that delayed confirmation of cancer diagnosis elevates patient anxiety and distress.^19^ In response to these concerns, numerous European countries and the USA have published organizational guidelines featuring recommended diagnostic and treatment intervals. Notable organizations like the British Thoracic Society (BTS), the National Institute for Health and Care Excellence (NICE), Swedish and Danish Lung Cancer Groups (SLCG, DLCG), the American College of Chest Physicians (ACCP), and the Institute of Medicine (IOM) have all contributed to establishing these crucial benchmarks.

The delays in diagnostic timelines represent a well-recognized and pervasive issue within the healthcare systems. Numerous medical centres have reported that diagnostic and treatment intervals frequently exceed the recommended timeframes for a significant portion of their patients. Addressing these challenges necessitates comprehensive improvements in the care pathways for lung cancer patients across various dimensions. Previous studies have consistently identified several common factors contributing to diagnostic delays, including prolonged waiting times for diagnostic procedures, multiple attempts required to establish a diagnosis, limited access to high-yield investigations, delays in staging procedures, and protracted turnaround times for results.^20,21,22,23^ Effective strategies to mitigate these delays have included the establishment of rapid access clinics designed to streamline the coordination of diagnostic procedures, the implementation of structured cancer diagnostic pathways, and the initiation of quality improvement projects that have successfully reduced redundant investigations and unnecessary inpatient admissions.^14,24,25,26,27^ An intriguing study conducted in Texas employed an electronic medical record trigger system to prospectively identify patients at risk of experiencing delays in their diagnostic evaluations.^28^

In our specific case, the prolonged waiting time for the initial clinic examination emerged as the primary culprit behind diagnostic delays, primarily due to the constraints posed by limited hospital capacities for lung cancer patients. Furthermore, the extended waiting times for PET-CT scans significantly contributed to the overall time required for arriving at a final diagnosis. Considering that the majority of our patients presented with nonmetastatic disease and approximately two-thirds of them necessitated a PET-CT scan before treatment decision could be made, it becomes less surprising that the time to reach a final diagnosis often exceeded the recommended 31-day threshold. An analysis encompassing 39 and 38 patients referred during two distinct periods, January and June 2023, substantiated the critical role of the factors mentioned above. A minor clinic reorganization undertaken in the spring resulted in increased capacities for managing lung cancer patients, leading to an average reduction of 4 days in the time interval from GP referral to the initial clinic examination. Moreover, this reorganization enabled 60% of patients to undergo their first assessment at the clinic within the recommended 14 days. Additionally, the notable decrease in the average waiting time for PET-CT scans in June, which was nearly 9 days, could be attributed to the acquisition of a new PET-CT machine. Collectively, these two strategic changes significantly shortened the overall time required to reach a final diagnosis by an average of 12.7 days.

Previous retrospective study from our clinic reported that 61 patients with lung cancer were diagnosed out of 159 patients who were examined in 2008 in specialized out-patient clinic for lung lesions in 12 months. The authors did not check the time from GP referral to the first visit, but reported the median time from the onset of symptoms to the first visit in outpatient clinic, which was 67 days. The median time from the first visit at clinic to the diagnosis was 10 days and from diagnosis to the beginning of treatment of 12 days. The important difference between the two analysed groups was a proportion of patients in whom PET CT was mandatory prior to treatment decision – 77% of patients in current study were of stage I–III and needed PET CT in comparison with less than half in 2008.^29^

Patients expressed high levels of satisfaction throughout every stage of the diagnostic pathway, spanning from the initial GP referral to the receipt of a final diagnosis. Surprisingly, more than half of the patients reported minimal stress during the diagnostic period. Those who did experience stress typically attributed it to concerns related to their diagnosis, personal or environmental factors. Notably, less than 10% felt stressed due to extended waiting times, and less than 5% cited a lack of information as a stress-inducing factor. These findings are particularly noteworthy in light of a previous study that highlighted the frustration experienced by patients and GPs when faced with undefined diagnostic pathways leading to respiratory specialist assessments and hospital clinics.^13^ It appears that the presence of a uniformly organized and well-coordinated pathway played a pivotal role in both the high levels of patient satisfaction and the low reported stress levels. Within this structured pathway, patients received holistic management and guidance from a single center, which facilitated appointment scheduling for all necessary investigations, managed test results, and maintained regular communication with patients. To further enhance the patient experience and alleviate anxiety, the implementation of automated notifications, such as emails or SMS messages, to keep patients informed about their progress along the diagnostic journey is recommended. Such a system could also reduce the volume of incoming patient inquiries regarding results and appointment status.

Although medical staff have expressed limitations in their available time for patients and caregivers interactions, patients generally reported receiving sufficient information about their medical management from the healthcare team. Nevertheless, we believe that the creation and distribution of an informational brochure or an electronic application (e-application) containing comprehensive details about the patient’s journey, including individual stages and diagnostic procedures, could further alleviate patient anxieties. Such printed resource may particularly benefit individuals who prefer offline, easily accessible information, reinforcing an inclusive approach to patient education. Moreover, the integration of Patient Reported Outcomes Measures (PROMs) and Patient Reported Experience Measures (PREMs) should be incorporated into routine practice. These measures offer a holistic assessment of care quality from the patient’s perspective, fostering a patient-centric healthcare culture and nurturing a feedback-based continuous improvement approach.^30^

While our study provides valuable insights, it is important to recognize its limitations. We conducted our research at a single center within the country, limiting the generalizability of our findings to a national level. Additionally, our study was limited in scope due to a relatively short timeframe. To ensure consistent treatment for patients with suspected lung cancer nationwide and to gain a comprehensive understanding of the issue, further research at a national level is needed. This broader investigation would address the specific needs and challenges faced by patients in various regions, ultimately enhancing the quality of care and outcomes for individuals with suspected lung cancer on a national scale.

## Conclusions

Our study, employing a comprehensive methodology, not only gathered insights from healthcare professionals involved in the diagnostic pathway but also incorporated valuable perspectives from patients themselves. This multifaceted approach provided a deep understanding of the diagnostic patient journey and served as a foundation for developing customized strategies for improvement. While it is evident that the patient journey from GP referral to MDTB treatment decisions is well-structured, coordinated, and garners high levels of patient satisfaction, our survey has uncovered critical areas requiring enhancement. Foremost among these is the development of an Electronic Diagnostic Clinical Pathway (eDCP), a pivotal initiative that can significantly enhance the process by alleviating unnecessary administrative burdens on staff. Moreover, it can provide a secure and reliable checklist and analytical system for regular process evaluations, ensuring ongoing improvements. On a systemic level, it is imperative to further bolster clinic capacities dedicated to patients with lung cancer. In particular, national investments in additional PET-CT machines, accompanied by the necessary medical personnel, are urgently needed to expedite the diagnostic process. In sum, this study illuminated opportunities for refining the diagnostic journey for lung cancer patients, underscoring the importance of both administrative and capacity-related enhancements.

## Supplementary Material

Supplementary Material Details
