# Supplementary material for: Unravelling the lung cancer diagnostic pathway: identifying gaps and opportunities for improvement
Source: Radiol Oncol. 2024 Apr 14;58(2):268–78. doi: 10.2478/raon-2024-0025 (PMC11165972; doi:10.2478/raon-2024-0025)
Supplement: Supplementary file 1 — Supplementary Material Details [file raon-2024-0025-sm.pdf]

Unravelling the lung cancer diagnostic pathway: identifying gaps and opportunities for improvement

Mateja Marc Malovrh, Katja Adamic

doi: 10.2478/raon-2024-0025

SUPPLEMENTARY TABLE 1. Questionnaire for hospital staff. [Translated from Slovenian]

**With this questionnaire, we want to better understand the current situation on the diagnostic path of a patient with suspected lung cancer. Based on your answers, we want to further improve this patient journey both from the perspective of the work of the hospital staff and from the perspective of better patient care.**
Thank you in advance for your answers.
THE QUESTIONNAIRE IS ANONYMOUS
**I work as:**
• Administrator
• Ordering Coordinator
• Radiological engineer
• Graduate nurse / medical technician
• Intermediate Nurse / Medical Technician
• Radiologist / Radiologist
• Doctor
**What would you point out as a positive aspect of your job?**
Your reponse
**Do you feel stressed at work?**
• YES
• NO
**Do you feel stressed at work?**
• YES
• NO
**How would you rate the level of stress at work?**
• Mild
• Moderate
• Horrible
• Extreme
**What would you single out as the most stressful aspect of your job?**
Your response
**Please rate**
• I do not agree at all
• I do not agree
• I am neutral
• I agree
• I agree very much

**With this questionnaire, we want to better understand the current situation on the diagnostic path of a patient with suspected lung cancer. Based on your answers, we want to further improve this patient journey both from the perspective of the work of the hospital staff and from the perspective of better patient care.**
I have too much work
I don't have enough work
My work is repetitive and monotonous
I don't have enough time to finish the work
I have enough breaks during work
I don't have enough time for my hobbies
I have very long working hours
I have too much work
I don't have enough work
My work is repetitive and monotonous
I don't have enough time to finish the work
I have enough breaks during work
I don't have enough time for my hobbies
**Please name three things that you would like to change and that would help you cope with everyday work and stress?**
Your response
**Very briefly describe in which procedures you are involved in the diagnostic process of a patient with suspected lung cancer.**
Your response
**Please rate**
• 1 very bad
• 2
• 3
• 4
• 5 very good
How well defined are the work processes related to diagnosing lung cancer?
How well is the planning of diagnostic tests and procedures optimized?
How well are patients guided throughout the treatment journey?
How well do the different healthcare teams within the hospital communicate with each other?

With this questionnaire, we want to better understand the current situation on the diagnostic path of a patient with suspected lung cancer. Based on your answers, we want to further improve this patient journey both from the perspective of the work of the hospital staff and from the perspective of better patient care.

How well are the processes for the preparation of councils organized?

How well are the processes for the preparation of councils organized?

How well would you rate the performance of the hospital information system?

How well do you think the patient documentation is organized?

How well are the processes related to managing the patient record organized

How well is clinical data collection organized in your department?

How effectively do current protocols ensure data security?

How well defined are the work processes related to diagnosing lung cancer?

How well is the planning of diagnostic tests and procedures optimized?

How well are patients guided throughout the treatment journey?

How well do the different healthcare teams within the hospital communicate with each other?

How well are the processes for the preparation of councils organized?

How well would you rate the performance of the hospital information system?

How well do you think the patient documentation is organized?

How well are the processes related to managing the patient record organized?

How well is clinical data collection organized in your department?

How effectively do current protocols ensure data security?

Is sufficient psychological support available to patients during treatment?

- YES
- NO

Are there bottlenecks in the patient journey that could lead to potentially avoidable patient delays and backlogs?

- NO
- YES

With this questionnaire, we want to better understand the current situation on the diagnostic path of a patient with suspected lung cancer. Based on your answers, we want to further improve this patient journey both from the perspective of the work of the hospital staff and from the perspective of better patient care.

If YES. Please highlight the bottlenecks?

Your response

Are there capacity issues related to staff, equipment, facilities in the path of a patient with suspected lung cancer?

- NO
- YES

If YES. Please list the most important problems.

Your respone

Please rate:

- 1 very bad
- 2
- 3
- 4
- 5 very good

How well are patients informed about lung cancer (symptoms, causes, complications, etc.)?

How well are patients informed about the diagnostic procedures they need to undergo before a final diagnosis is made?

How well are patients informed about their diagnosis (disease stage, factors, risk, etc.)?

How well are patients informed about possible lifestyle changes as a way to manage the disease?

How well are patients informed about lung cancer (symptoms, causes, complications, etc.)?

How well are patients informed about the diagnostic procedures they need to undergo before a final diagnosis is made?

How well are patients informed about their diagnosis (disease stage, factors, risk, etc.)?

How well are patients informed about possible lifestyle changes as a way to manage the disease?

Do you have any other comments, suggestions or ideas that would contribute to improving the treatment of patients with suspected lung cancer?

Your response

Send

SUPPLEMENTARY TABLE 2. Questionnaire **Q1** pre-visit to the clinic. [Translated from Slovenian]

**This survey is designed to gain a comprehensive understanding of the current diagnostic pathway for patients with suspected lung cancer. Through your insights, we aspire to enhance this pathway, optimizing both the efficacy of hospital personnel and the quality of patient care.**
We extend our sincere gratitude for your forthcoming responses
THE QUESTIONNAIRE IS ANONYMOUS
**Into which age bracket do you fall?**
• Up to 40 years
• 40 to 60 years
• 60 to 80 years
• Over 80 years
**How would you evaluate your satisfaction with the pre-arrival treatment at Clinic Golnik?**
• 1 Unsatisfactory
• 2
• 3
• 4
• 5 Very satisfactory
**What duration transpired between the referral by your primary care physician and admission to the Clinic Golnik?**
• 1 week
• 1 to 2 weeks
• 2 to 3 weeks
• 3 weeks or more
**How promptly were you notified of the examination date?**
• Within 3 days
• 3 to 7 days
• More than 7 days

**This survey is designed to gain a comprehensive understanding of the current diagnostic pathway for patients with suspected lung cancer. Through your insights, we aspire to enhance this pathway, optimizing both the efficacy of hospital personnel and the quality of patient care.**
**How content are you with the procedure and methodology of scheduling an examination at Clinic Golnik?**
• 1 Unsatisfactory
• 2
• 3
• 4
• 5 Very satisfactory
**Did you receive unequivocal instructions regarding the procedure and available options for scheduling an examination at Clinic Golnik?**
• YES
• NO
• Instructions could have been more explicit
**While scheduling an examination, were you provided with clear directives on prerequisites before the examination and the items to bring along (test results, images, medications, etc.)?**
• YES
• NO
• Instructions could have been more explicit
**Did you identify any informational gaps during scheduling and the waiting period for the examination?**
Your response
**Would you be able to propose enhancements to the scheduling process?**
Your response
**Send**

SUPPLEMENTARY TABLE 3. Questionnaire **Q2.** It completed by the patients after the outpatient clinic visit. [Translated from Slovenian]

**With this questionnaire, we want to better understand the current situation on the diagnostic path of a patient with suspected lung cancer. Based on your answers, we want to further improve this patient journey, both from the point of view of the work of the hospital staff and from the point of view of better patient care.**	
Thank you in advance for your answers.	
THE QUESTIONNAIRE IS ANONYMOUS	
**Please rate how difficult it was for you to find your way to the right clinic and laboratories within the hospital?**	
• 1	Very difficult
• 2	
• 3	
• 4	
• 5	Very easily
**How long did you wait in the waiting room before being admitted to the outpatient clinic for pulmonary infiltrates?**	
• Less than 15 min	
• 15–30 min	
• 30–45 min	
• more than 45 min	
**Please rate how clear the information and instructions you received during the treatment at the outpatient clinic for lung infiltrates were.**	
• 1	The information was not clear
• 2	
• 3	
• 4	
• 5	The information was very clear
**Did you receive all the necessary information you needed from the hospital staff?**	
• YES	
• NO	
**If NOT. What additional information would you like?**	
Your answer	

**With this questionnaire, we want to better understand the current situation on the diagnostic path of a patient with suspected lung cancer. Based on your answers, we want to further improve this patient journey, both from the point of view of the work of the hospital staff and from the point of view of better patient care.**	
**Rate how well you were informed about the steps/ examinations that you still have to do before the doctor can make a correct diagnosis?**	
• 1	Very poor
• 2	
• 3	
• 4	
• 5	Very good
**Answer if you have been to an outpatient clinic for lung infiltrates:**	
**Were the instructions related to ordering a PET-CT examination clear enough?**	
• YES	
• NO	
• I would like more information	
**Answer if you have been to an outpatient clinic for lung infiltrates:**	
**Please rate how satisfied you were with the treatment at the outpatient clinic for lung infiltrates**	
• 1	I was not satisfied
• 2	
• 3	
• 4	
• 5	I was very satisfied
**Could you suggest any improvement in the treatment in the outpatient clinic for pulmonary infiltrates?**	
Your response	
**Send**	

SUPPLEMENTARY TABLE 4. Questionnaire **Q3.** After hospital discharge. [Translated from Slovenian]

**With this questionnaire, we aim to gain a comprehensive understanding of the current status along the diagnostic pathway for patients suspected of lung cancer. Based on your responses, our objective is to further enhance this pathway, both from the perspective of hospital staff and in terms of improving patient care.**	
Thank you in advance for your answers.	
THE QUESTIONNAIRE IS ANONYMOUS	
**How were you referred for hospital treatment?**	
• After examination in the outpatient clinic for lung infiltrates	
• Admission through the emergency outpatient clinic	
• Pre-scheduled admission date without an examination in the outpatient clinic	
**How long did you wait for admission to the hospital, or how many days elapsed from your examination in the outpatient clinic?**	
• Less than 1 week	
• 1 to 2 weeks	
• 2 to 3 weeks	
• More than 3 weeks	
**How long did you wait to be assigned a bed upon hospital admission?**	
• Less than 1 hour	
• 1 to 2 hours	
• More than 2 hours	
**Please assess how well you were informed about the steps and examinations that awaited you during hospitalization.**	
• 1	Very poor
• 2	
• 3	
• 4	
• 5	Very good
**During hospitalization, did you have enough time to talk with the healthcare staff?**	
• YES	
• NO	
**Did you receive all the necessary information from the hospital staff during hospitalization?**	
• YES	
• NO	
**If NOT. What other information would you like?**	
Your response	

**With this questionnaire, we aim to gain a comprehensive understanding of the current status along the diagnostic pathway for patients suspected of lung cancer. Based on your responses, our objective is to further enhance this pathway, both from the perspective of hospital staff and in terms of improving patient care.**	
**Please assess how well you understood the doctor's information about your preliminary diagnosis.**	
• 1	Very poor
• 2	
• 3	
• 4	
• 5	Very good
**Evaluate how often you received conflicting information from hospital staff.**	
• 1	Very often
• 2	
• 3	
• 4	
• 5	Never
**Please rate your satisfaction with the healthcare provided during your hospitalization.**	
• 1	Not satisfied
• 2	
• 3	
• 4	
• 5	Very satisfied
**Please assess how well you understood your next steps after discharge from the hospital.**	
• 1	Very poor
• 2	
• 3	
• 4	
• 5	Very good
**After discharge from the hospital, were you aware of whom to contact and how for any additional questions?**	
• YES	
• NO	
**Could you suggest any improvements to the healthcare provided during hospitalization?**	
Your response	
**Send**	

SUPPLEMENTARY TABLE 5. Questionnaire **Q4.** For patients after a multidisciplinary treatment board. [Translated from Slovenian]

**With this questionnaire, we aim to gain a deeper understanding of the current status along the diagnostic pathway for patients suspected of lung cancer. Based on your responses, our goal is to further enhance this pathway, both from the perspective of hospital staff and in terms of improving patient care.**	
Thank you in advance for your answers	
THE QUESTIONNAIRE IS ANONYMOUS	
**How did you learn about the opinion of the Multidisciplinary Treatment Board?**	
• Notification by phone	
• Personal conversation with a doctor	
• Received a written report	
**Were you satisfied with the way the diagnosis was communicated to you?**	
• YES	
• NO	
**If No, what would you have preferred?**	
Your response	
**After presenting at the Multidisciplinary Treatment Board, you received additional information and further instructions. Were these instructions clear?**	
• YES	
• NO	
**If No, what were you missing?**	
Your response	
**Please assess how well you understood what your next steps are in the treatment process?**	
• 1	Very poor
• 2	
• 3	
• 4	
• 5	Very good
**How long did the entire process take from your doctor's referral to receiving the Multidisciplinary Treatment Board opinion?**	
• Less than 2 weeks	
• 2 to 4 weeks	
• 4 to 6 weeks	
• 6 to 8 weeks	
• 8 to 10 weeks	
• More than 10 weeks	

**With this questionnaire, we aim to gain a deeper understanding of the current status along the diagnostic pathway for patients suspected of lung cancer. Based on your responses, our goal is to further enhance this pathway, both from the perspective of hospital staff and in terms of improving patient care.**	
**How many times did you visit Clinic Golnik before receiving the final diagnosis?**	
• 1 visit	
• 2 to 3 visits	
• More than 3 visits	
**Please rate your satisfaction with healthcare from your first contact with Clinic Golnik to receiving the final diagnosis.**	
• 1	Not satisfied
• 2	
• 3	
• 4	
• 5	Very satisfied
**What was the most stressful aspect for you during this period?**	
• Insufficient information	
• Long wait for diagnosis	
• Poor communication from hospital staff	
• Not stressful	
• Other:	
**Would you like more support from a psychologist?**	
• YES	
• NO	
**Do you believe your family members were adequately involved and informed about the diagnostic process?**	
• YES	
• NO	
• Other:	
**Could you possibly suggest any improvements in care during this period?**	
Your response	
**Send**	
**Clear the form**	
